# Well-being balance and lived experiences: understanding the impact of life situations on human flourishing

**DOI:** 10.3389/fpsyt.2024.1516729

**Published:** 2025-01-31

**Authors:** Christopher R. Brydges, Alexandra Thérond, Troy W. Norris

**Affiliations:** ^1^ NIH West Coast Center, University of California, Davis, Davis, CA, United States; ^2^ Department of Psychology, Université du Québec à Montréal, Montreal, QC, Canada; ^3^ WellBalance Institute, Boston, MA, United States

**Keywords:** subjective well-being, positive psychology, human flourishing, balanced well-being, well-being assessment, mindset, positivity, life situations

## Abstract

**Background:**

This study aimed to determine the most significant indicators of positive well-being and understand differences in sources of well-being across different life situations, age groups, genders, and income levels, utilizing a novel measure of positive well-being, the Well-being Balance and Lived Experiences (WBAL) Assessment, which evaluates the frequency of various positive experiences and feelings across a range of activation and arousal levels that have previously been demonstrated to affect subjective well-being and human flourishing.

**Methods:**

A sample of 496 evaluable subjects aged 20-69 and census-balanced for gender were recruited from a U.S. population panel. Differences in well-being and sources of well-being were analyzed across subgroups via MANOVA analysis followed by *post-hoc* ANOVA and Tukey’s HSD analyses using Cohen’s *d* to determine size and direction of effects between categorical subgroups.

**Results:**

Life situations, including relationship, parenting and employment status, were shown to have a more significant effect on overall well-being than the demographic variables of age, gender and household income. Reported well-being improved significantly with life situations, including companionate relationships (*d*=0.38, *p*<0.001) and parenting (*d*=0.35, *p*<0.001), that provide greater opportunities for more frequent social connection (*d*’s=0.25, *p*<0.01 to 0.62, *p*<0.001) and purposeful contribution to others’ well-being (*d*’s=0.34 to 0.71, *p*<0.001), associated with increased feelings of significance (*d*’s=0.40 to 0.45, *p*<0.001) and efficacy (*d*’s=0.37 to 0.44, *p*<0.001). An age-related positivity effect was observed, with older adults reporting more frequent positive feelings than younger age groups (*d*=0.31, *p*<0.01). Measures of mindset positivity, variety of positive experiences and feelings, and frequency and range of positive feelings across arousal levels each corresponded closely with overall well-being.

**Conclusion:**

Life situations, including relationship, parenting and employment status, had a more broad and significant effect on wellbeing than age, gender or income. Across life situations, purposeful contribution and social connection, with associated feelings of efficacy and significance were key drivers of differences in well-being. Mindset positivity and variety of positive experiences and feelings correspond closely with overall well-being. Findings from this study can help guide the design and implementation of intervention programs to improve well-being for individuals and targeted subgroups, demonstrating the utility of the WBAL Assessment to evaluate discrete modifiable sources of positive well-being.

## 1 Introduction

Understanding what drives human flourishing, subjective well-being, overall life satisfaction and positive psychological functioning is crucial for direct public health initiatives, social policy and individual wellbeing interventions. Research has demonstrated that these measures of well-being vary systematically across populations with different demographic characteristics and life circumstances, including age, gender, annual household income, relationship status, parenting status, and employment status.

For example, age has consistently been shown to have a U-shaped relationship with subjective well-being, where life satisfaction reaches its lowest point between the mid-30s and mid-50s (1). While recent research has identified several age-related psychological and social factors that contribute to well-being (2), such as the tendency toward more positive emotional experiences in later life (3, 4), the comprehensive factors underlying the relationship between well-being and age remain incompletely understood.

Gender differences in well-being also present a nuanced picture. While men and women report comparable levels of overall life satisfaction and affect balance (5), they differ in their sources of well-being. For example, men show consistently higher engagement in physical activity (6–8), whereas women have stronger positive relations with others (9). These gender-specific pathways to well-being illustrate how different individuals can achieve similar levels of life satisfaction in different ways.

Income represents another key demographic factor, showing a consistent but complex relationship with well-being. Within the US, both immediate positive emotions and overall life satisfaction increase with income, following a log-linear pattern, with progressively smaller improvements in well-being as baseline income rises (10–13). Elucidating the underlying drivers of this complex relationship between economic resources and psychological flourishing has important implications for understanding how socioeconomic factors influence well-being across different population segments.

Relationship status has been shown to have a strong influence on well-being, with more committed relationships generally associated with greater life satisfaction. This forms a clear hierarchy: married individuals report the highest well-being, followed by those in cohabiting relationships, steady dating relationships, casual dating relationships, and finally those who date infrequently or not at all (14). Marriage has been shown to be particularly beneficial, providing both immediate (15, 16) and sustained long-term improvements in well-being (17, 18). Notably, married couples report more stable life satisfaction through middle age compared to their unmarried counterparts (19).

Parenting represents another significant life role that influences well-being with multifaceted effects. Parents generally report higher levels of life satisfaction than non-parents (20), with benefits extending beyond happiness to include enhanced sense of meaning, positive emotions, and expanded social roles, relative to non-parents (21, 22).

Employment status shows a clear relationship with well-being, with distinct patterns across different working arrangements. Full-time employment is generally associated with the highest levels of well-being among the employed, while non-standard arrangements such as part-time and self-employment correspond with lower subjective well-being (23). Unemployment has particularly severe effects on well-being, creating a challenging cycle: the combination of psychological stress and financial strain reduces mental health (24), which in turn can make finding and maintaining employment more difficult (25). This reciprocal relationship between employment and well-being highlights how life circumstances can create self-reinforcing patterns of psychological flourishing or distress.

While these demographic and life circumstance indicators consistently predict well-being, their relative magnitude of effect and complex underlying mechanisms are not fully understood. Understanding the complete picture requires examining the underlying experiential and emotional sources that contribute to well-being across demographic groups. These sources can be broadly categorized into several domains: physical and mental wellness, social connection and openness, purposeful experiences, and feelings of significance and efficacy. By examining how these fundamental sources of well-being vary across different population groups, we can better understand the mechanisms through which demographic factors influence life satisfaction.

Within the domain of physical and mental wellness, several key behavioral factors emerge as fundamental to well-being. Physical activity shows particularly robust effects, with increases in exercise consistently leading to improvements in happiness, positive affect, life satisfaction, and self-esteem (26, 27). Eating nutritiously is important for maintaining psychological well-being (28, 29), and sufficient sleep is essential for emotional and physical wellness (30).

Beyond physical wellness, engagement in cognitively and emotionally enriching and soothing activities plays a crucial role in well-being. These activities span a wide range, from creative expression (31) and flow experiences (32), to being exposed to nature (33, 34), appreciating art (35), and musical engagement (36). Each of these experiences contribute to well-being through distinct but complementary psychological mechanisms, suggesting that mental enrichment represents a fundamental pathway for flourishing.

Feelings of meaning, significance and mattering are also central sources of well-being, that are closely interconnected with social relationships (37). Beyond simply contributing to well-being and health (38), feelings of meaning and significance serve as a psychological resource, helping individuals maintain positive mental health even in the face of life stressors (39). This protective effect appears particularly linked to positive social connections, though the benefits of social relationships depend on both their quantity and quality: both the number of social connections and their perceived supportiveness contribute to well-being over time (40).

Diversity of positive experiences and feelings is another crucial dimension that increases subjective well-being, flourishing and resilience. Feeling a rich and varied range of positive emotions, such as joy, gratitude, serenity and pride, enhances psychological and physical well-being (41). This relationship appears to operate through daily experiences, as greater emotional variety in response to everyday positive events predicts higher levels of subjective well-being (42). Intentionally participating in a variety of positive activities leads to a broader range of positive emotions, even without changes in life circumstances (43).

Engaging in a wider range of enjoyable activities with varying activation levels is associated with improved psychosocial and physical well-being (44). And hedonic adaptation – the tendency for the degree of happiness derived from the same positive experiences to decrease with repetition – can be prevented by increasing the variety of positive experiences, along with increased appreciation of these positive experiences through savoring and gratitude (45, 46).

The relationship between emotional variety and well-being may also be explained in part through the broaden-and-build theory, which describes a self-reinforcing cycle whereby diverse positive emotions encourage exploration of novel experiences, which in turn generate more positive emotions, ultimately building psychological resilience and stress-coping capacity in an upward spiral of positivity (47, 48).

Recent research has demonstrated the particular importance of lower arousal positive emotional states for well-being. For example, contentment has been shown to be an important determinant of well-being and life satisfaction (49), and dispositional mindfulness and serenity are associated with lower stress and increased mental well-being (50). Individuals who more frequently feel positive emotions spanning from low to high arousal levels, are better able to cope with stress and respond to adverse situations (51).

Increasing mindset positivity also appears to be an important factor in overall well-being, with multiple studies demonstrating independent associations of well-being with mindful positivity practices, such as savoring, gratitude and compassion. Increased savoring of positive experiences has been demonstrated to improve subjective well-being (52), including increasing happiness (53), and life satisfaction (54). Gratitude has been shown to be positively associated with subjective well-being (55, 56), as have loving kindness and compassion (57).

This study aims to deepen the understanding of well-being variations across population subgroups by determining the most significant indicators of well-being – including life situations, age, gender, and income – and evaluating changes in specific sources of well-being across these indicators. This study utilizes the Well-being Balance and Lived Experiences (WBAL) Assessment, shown in **Table 1**, a comprehensive measure of positive well-being that evaluates multiple sources of well-being simultaneously (58), to explore the impact of differing well-being indicators on overall well-being and underlying sources of well-being.

**Table 1:** Well-being and lived experiences assessment instrument, 30-item (WBAL-30).

Domain	Factor	Energy Level	Item	#	Prompt
Experiences		Activation Level:			Over the past two weeks, how often have you had the following experiences? (0 = Rarely, 1 = Sometimes, 2 = Often, 3 = Usually, 4 = Very Often)
	Body	Active	Move Regularly	1	My days are physically active, I exercise regularly, and my body is strong and able.
		Mindful	Nourish Healthily	2	I savor nutritious food and eat only until full, while hydrating regularly without too much alcohol or caffeine.
		Calm	Rest and Recover	3	I sleep well and let myself rest and recover when I’m sore, injured or tired.
	Mind	Active	Create, Learn and Explore	4	I learn new things, express my creativity and become fully absorbed in activities.
		Mindful	Savor and Appreciate	5	I spend time in nature, and appreciate and enjoy music, art, and good stories.
		Calm	Reflect Gratefully	6	I pause to reflect, feel grateful and connect to something larger than myself.
	Connection	Active	Build Community	7	I engage with groups beyond my close friends and family, and seek out new people that share my interests.
		Mindful	Bond Closely	8	I regularly connect with my close friends or family and we help each other when needed.
		Calm	Love Securely	9	I spend undistracted time with a loving, trusted companion, and we listen to and meet each other’s needs.
	Purpose	Active	Contribute, Serve and Earn	10	I help make the world better, positively impact others, and am rewarded fairly for my work.
		Mindful	Provide and Nurture	11	I am responsible, provide for others’ wellbeing and help make my home comfortable and safe.
		Calm	Kindness and Grace	12	I am kind to others, supporting and comforting them, without judgment or resentment.
	Activation Range	Active	Active and Engaged	13	My body is active and fit, my mind is engaged, and I have a meaningful impact in my community
		Mindful	Mindful and Present	14	I pay attention to and take care of myself and others, am present in the moment and appreciate the world around me.
		Calm	Calm and Restful	15	My relationships are secure, I am physically safe, and I can relax and be at peace.
Feelings		Arousal Level:			Over the past two weeks, how often have you had the following feelings? (0 = Rarely, 1 = Sometimes, 2 = Often, 3 = Usually, 4 = Very Often)
	Arousal Range	Joyful	Joyful and Confident	16	My life feels meaningful and fun, filled with purpose, joy and laughter.
		Aware	Aware and Appreciative	17	I savor life’s special moments, am self-aware, and appreciate the people in my life.
		Content	Content and Peaceful	18	I feel content and satisfied with my life, at peace with myself and safe with others.
	Openness	Joyful	Adventurous and Curious	19	I enjoy meeting new people, exploring new cultures and trying new experiences.
		Aware	Harmonious and Attentive	20	I appreciate nature, art and music, and feel connected to people in my life and in harmony with my world.
		Content	Trusting and Safe	21	I trust myself and others to keep us safe, and believe things will work out.
	Significance	Joyful	Proud and Mattering	22	My life matters and has meaning, and I am proud of my accomplishments.
		Aware	Belonging and Accepted	23	I feel like I belong, am welcome and appreciated, and can be myself with people in my life.
		Content	Gentle and Loved	24	I feel loving kindness and am gentle towards others, and feel loved and cared for in return.
	Efficacy	Joyful	Capable and Confident	25	I feel confident and capable to contribute meaningfully and take care of myself and others.
		Aware	Considerate and Responsible	26	Others can depend on me and I feel able to provide for myself and others.
		Content	Caring and Compassionate	27	I care for and feel compassion towards myself and others.
	Wellness	Joyful	Vital and Strong	28	I feel alive and energetic, with a strong body and sharp mind.
		Aware	Satisfied and Fulfilled	29	I feel fulfilled and satisfied, appreciating small pleasures in the moment.
		Content	Peaceful and Serene	30	My life feels peaceful, serene and untroubled, with a restful body and calm mind.

Copyright WellBalance, LLC (2023); available for research use with permission.

## 2 Materials and methods

### 2.1 Materials

The study employs the Well-being Balance and Lived Experiences Assessment, 30-item (WBAL-30). The WBAL Assessment is a validated measure of positive well-being that evaluates the self-reported frequency of twenty-four (24) distinct categories of positive experiences and feelings previously demonstrated to be associated with positive well-being. These categories include physical and mental wellness, social connection and openness, purposeful experiences, and feelings of significance and efficacy. The instrument also includes measures of the breadth of sources of well-being, mindset positivity and the frequency and range of experience activation levels and feeling arousal levels.

The WBAL Assessment has been demonstrated to be a reliable and valid instrument to comprehensively measure positive aspects of well-being and evaluate multiple modifiable sources of individuals’ well-being (58). A confirmatory factor model showed good fit, indicating that each of the model factors are related but distinct and all items load significantly onto their factors. The WBAL Assessment demonstrated high internal consistency (Cronbach’s α = 0.95) and internal validity across well-being factors and Feelings (r = 0.96) and Experiences (r = 0.94) domains. Furthermore, the WBAL Assessment demonstrated strong convergent validity in comparison to the PERMA+ Profiler (59) developed at University of Pennsylvania’s Positive Psychology Center (r = 0.80) and WBA-24 (60) developed collaboratively by Harvard University’s Human Flourishing Program and the Institute for Healthcare Improvement (r = 0.75), indicating that the WBAL Assessment measures a similar overall concept of well-being and flourishing. Discriminant validity of WBAL factors was demonstrated for an average of 14.3 of 17 comparator domains.

The WBAL Assessment measures the frequency of distinct categories of positive experiences and positive feelings related to well-being in the WBAL Model (**Appendix A**). WBAL-30 comprises 30 items in a set order scored on a 5-point Likert Scale (from 0 to 4) measuring respondents’ self-reported subjective frequency of each category of positive Experiences and Feelings over the past two weeks (0 = Rarely, 1 = Sometimes, 2 = Often, 3 = Usually, 4 = Very Often).

WBAL scores represent averages of all items overall and within two domains of positive experiences and positive feelings. Twelve (12) categories of positive experiences are mapped to four (4) factors of mind, body, connection and purpose, in addition to three (3) items evaluating the frequency and range of activation levels of experiences, mapped to an activation range factor. Twelve (12) categories of positive feelings are mapped to four (4) factors of wellness, openness, significance and efficacy, in addition to three (3) items evaluating the frequency and range of arousal levels of feelings, mapped to an arousal range factor.

As with the comparator scales, PERMA+ and WBA-24, there are not strictly defined thresholds for classifying low, moderate, and high well-being, due to the subjective nature of well-being and variations across different populations samples. Unlike these comparator measures, WBAL has a near-normal distribution on a scale from 0 to 4, with an average score of 2.3, a standard deviation of 0.8, and a median score of 2.4 with 25^th^ and 75^th^ percentile scores of 1.8 and 2.9, respectively.

### 2.2 Procedure and participants

Study participants were recruited in the United States by Momentive.ai from SurveyMonkey Audience panels. These panels are proprietary and exclusive, constituting a diverse group of people generally reflective of the US population. The panels are regularly calibrated to ensure high response quality, and members of whom have opted in to participate in research projects. Payment for participation was set and provided by Momentive and was set to be a nominal amount less than one US dollar ($1) per respondent.

Participants were required to be between the ages of 20 and 69 and have a minimum annual household income of $25,000 per year. Individuals with lower income and of higher age were excluded due to concerns about data quality and precision, due to educational and psychosocial factors (61) and potential sampling bias due to the digital survey methodology (62).

A total of 33 questions from the study survey instrument were used, including the WBAL-30 items and 3 questions regarding employment, relationship and parenting status. Additional demographic information provided by Momentive included respondents’ age, annual household income and geographic region in which they reside.

The study was determined by Solutions IRB to present no or minimal risk to research subjects and therefore exempt from ongoing IRB oversight. A statement of voluntary consent appeared at the beginning of the survey explaining that the subject’s participation is completely voluntary, they are not expected to benefit from participating, and they can stop participating at any time during the survey.

We planned to have 500 participants complete the survey. For initial MANOVA analyses with α=0.05, this total sample size provides >99% power to detect differences with a medium effect size (Cohen’s *f*) of 0.25 with up to 7 groups (the maximum compared) and up to 30 dependent variables (all WBAL items). For subsequent ANOVA analyses with α=0.05, this total sample size provides >99% power to detect differences with effect size (Cohen’s *d*) of 0.20 between two groups and >95% power between seven groups.

Additionally, to support exploratory analyses, the sample was stratified in a nested fashion across age groups and gender, with a target of 100 respondents census-balanced for gender in each age group of 20-29, 30-39. 40-49, 50-59, 60-69 years, providing 60% power of a two-tailed t-test to detect differences with effect size (Cohen’s *d*) of 0.20 between any two subgroups stratified by age and gender.

### 2.3 Analysis

All analyses were conducted in R 4.4.0. Overall items contributing to WBAL scores were assessed using one-way ANOVAs or one-way MANOVAs based on the average scores for each factor and item, representing the frequency of positive Experiences or Feelings. For each subgroup, mean scores were calculated for the overall instrument, and for each domain, factor and individual item within each assessment. Specifically, one-way MANOVAs were conducted on the WBAL overall score, domains, factors, and individual items, with *post-hoc* ANOVAs conducted if the omnibus MANOVA was statistically significant. If any ANOVA was significant, Tukey’s HSD test was conducted for pairwise comparisons, with Cohen’s *d* calculated as a measure of effect size. An alpha level of α = 0.05 was used for this study, and interpretation guidelines for Cohen’s *d* (63) were used, where effects of *d* = 0.20, 0.50, and 0.80 were considered to be small, medium, and large effects, respectively.

**Table 2** shows the logical nesting of independent variable categories applied for the MANOVA analysis. For independent variables, participants were binned into one of four age groups: 18-29 years (“Young Adults”), 30-44 years (“Established Adults”), 45-60 years (“Midlife Adults”), or 61+ years (“Older Adults”). Income was coded into bins of $25,000-$50,000 (“Lower Income”), $50,001-$75,000 (“Middle Income”), $75,001-$100,000 (“Higher Income”), and $100,001+ (“Highest Income”). Other independent variables either were not altered from the response options provided in the survey, or are self-explanatory (e.g., being a parent vs. being childfree).

**Table 2:** MANOVA *F*-values and significance level (*p*) for WBAL overall, domains, factors and individual items (*p<0.05, **p<0.01, ***p<0.001) by well-being indicator nested categories and subcategories.

Well-being Indicators	Category Comparisons	Subcategory Comparisons	Dependent Variables			
			WBAL	Domains	Factors	Items
Gender	Female vs. male		0.002	0.009	1.590	1.961 **
Age Range	Older adults vs. all other ages		7.392 **	4.315 *	1.728	1.427
		*Young vs. established vs. midlife adults*	0.042	0.803	1.479	1.701 **
Annual Household Income	Lower vs. middle vs. higher vs. highest income		0.052	1.625	1.250	1.173
Employment Status	Employed vs. student vs. unemployed vs. retired/homemaker		3.547 *	1.941	1.954 **	1.693 ***
	*Employed:*	*Employed full-time vs. part-time vs. self-employed*	1.16	1.660	1.455	1.275
	*Choosing to* *not work:*	*Retired vs. homemaker*	0.00	0.701	0.748	1.044
Relationship Status	Coupled vs. uncoupled		13.54 ***	7.845 ***	6.568 ***	6.450 ***
	*Coupled:*	*Steady relationship vs. living together vs. married*	3.101 *	1.609	1.792 *	1.293
	*Uncoupled:*	*Single vs. divorced vs. widowed*	1.13	0.557	0.582	0.687
Parenting Status	Parents vs. no children		14.73 ***	11.204 ***	4.579 ***	2.689 ***
	*Parents:*	*Active parents vs. children not home*	2.068	1.306	1.627	1.600 *
	*Active parents:*	*Single-parent vs. co-parenting vs. two-parent household*	0.882	0.482	1.024	1.365 *

## 3 Results

Mean scores for population subgroups, including overall WBAL scores, mindset positivity, number of frequent well-being sources, domains, factors and individual items can be found in the **Supplementary Materials**. The **Supplementary Materials** also include *z*-score differences of subgroups from mean values, with *z*-test significance levels, as well as the results of all MANOVA analyses performed. **Table 2** shows the *F*-values and significance of MANOVA results for analyses at each hierarchical level, including WBAL overall, domains, factors and individual items. All MANOVA analyses were significant for comparisons of age ranges (older adults vs. all other younger age groups), overall employment status, overall relationship status (coupled vs. uncoupled) and overall parenting status (parents vs. no children). However, the significance levels of more detailed nested comparisons for employment, relationship and parenting status were generally lower. MANOVA analyses for gender were only significant for select individual items, and no MANOVA analyses were significant for household income.

### 3.1 Evaluable subjects

A total of 496 participants were included in the analyses as evaluable subjects. As shown in **Table 3**, the evaluable participants were 57% female, 53% employed full-time, 57% married or in a domestic partnership, 63% parents, and reported a median annual household income of $50,000 to $75,000. with 78% making less than $100,000 per year.

**Table 3:** Sample sizes by demographic category.

	Category	n	%
All Evaluable Subjects		496	100%
Gender Identification	Male	212	43%
	Female	284	57%
Age Range	Young Adults (20–29)	92	19%
	Established Adults (30–44)	151	30%
	Midlife Adults (45–60)	158	32%
	Older Adults (61–69)	95	19%
Annual Household Income	Lower Income ($25k-$50k)	141	28%
	Middle Income ($50k-$75k)	131	26%
	Higher Income ($75k-$100k)	117	24%
	Highest Income ($100k+)	107	21%
Employment Status	Unemployed (and seeking work)	10	2%
	Part-Time Employed	35	7%
	Self-Employed	41	8%
	Student	20	4%
	Full-Time Employed	265	53%
	Homemaker (not working outside home)	40	8%
	Retired	78	16%
Relationship Status	Single (not in a relationship)	80	16%
	Steady Relationship	37	7%
	Living Together	59	12%
	Married or Domestic Partnership	282	57%
	Divorced or Separated	24	5%
	Widowed	7	1%
Parenting Status	No Children	182	37%
	Single-Parent Household (primary caregiver)	28	6%
	Co-Parent (split time, custody arrangement)	13	3%
	Two-Parent Household	151	30%
	Parent with Children not Home	111	22%

Momentive automatically screened out potential bots or fraudulent responses based on email and location verification. Momentive also used ID exclusions to prevent duplicate responses. After this initial screening, there were 646 total respondents. Two responses were incomplete and excluded from analysis. An additional 33 responses were removed from the analysis as “cheaters” whose responses on survey questions were all within a tight range and had answers to reverse coded items in the same range, indicating that the respondents were not reading and accurately responding to specific questions. An additional 115 responses were identified as “speeders” based on completion times being less than 2/3 of the median completion time for the survey.

### 3.2 Well-being sources

**Figure 1:**
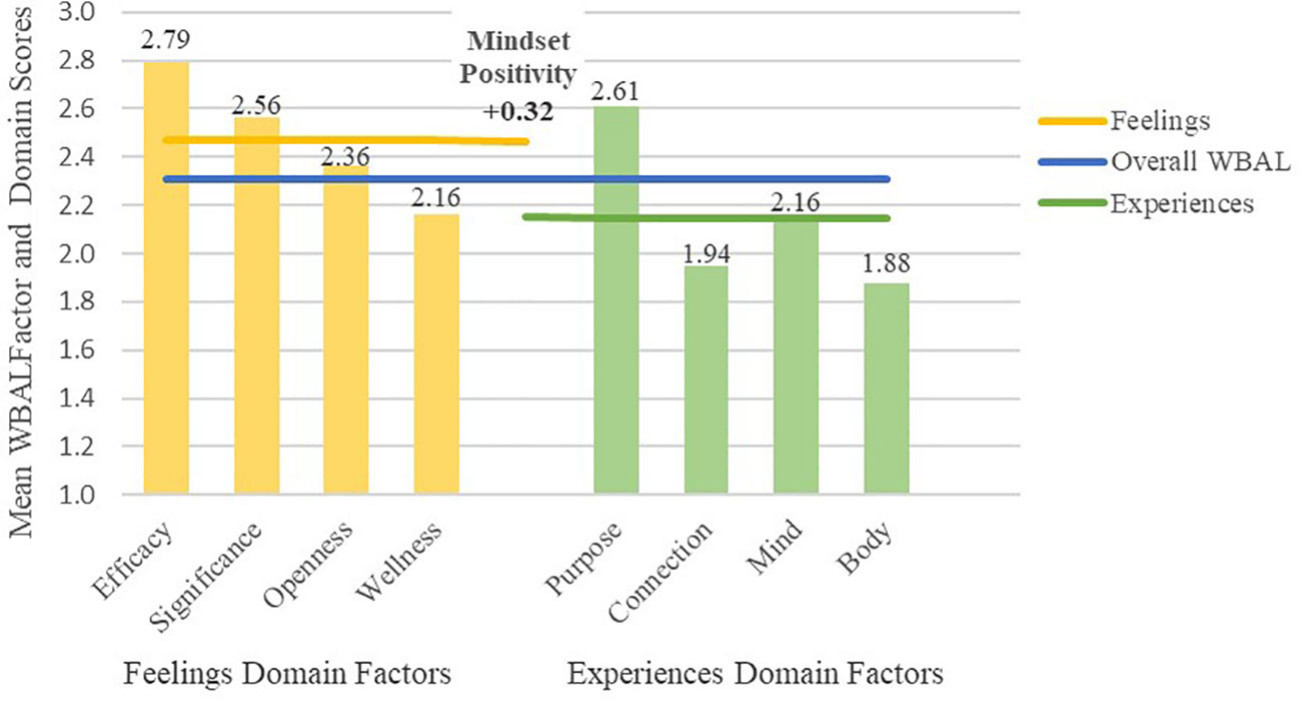
Mean WBAL scores by WBAL domain and factor for all respondents.

As shown in **Figure 1**, the most frequently reported positive experiences contributing to well-being among respondents were purposeful contributions, corresponding with frequent feelings of efficacy and significance. In contrast, respondents reported less frequent positive self-care experiences, corresponding with less frequent feelings of wellness, and less frequent social connection. On average, respondents reported having positive feelings more frequently than they engaged in positive experiences, indicating a generally positive mindset.

### 3.3 Indicators of positive well-being

Higher well-being was most broadly and significantly associated with being in a companionate relationship and being a parent across respondents. Retirees and homemakers reported higher well-being, whereas unemployment was associated with lower well-being. Well-being among older respondents was significantly higher than other age groups, with reported well-being being lowest among midlife respondents. There were no significant differences in overall well-being between genders or across income levels, although some differences in underlying sources of well-being were observed between groups.

#### 3.3.1 Relationship status

**Figure 2:**
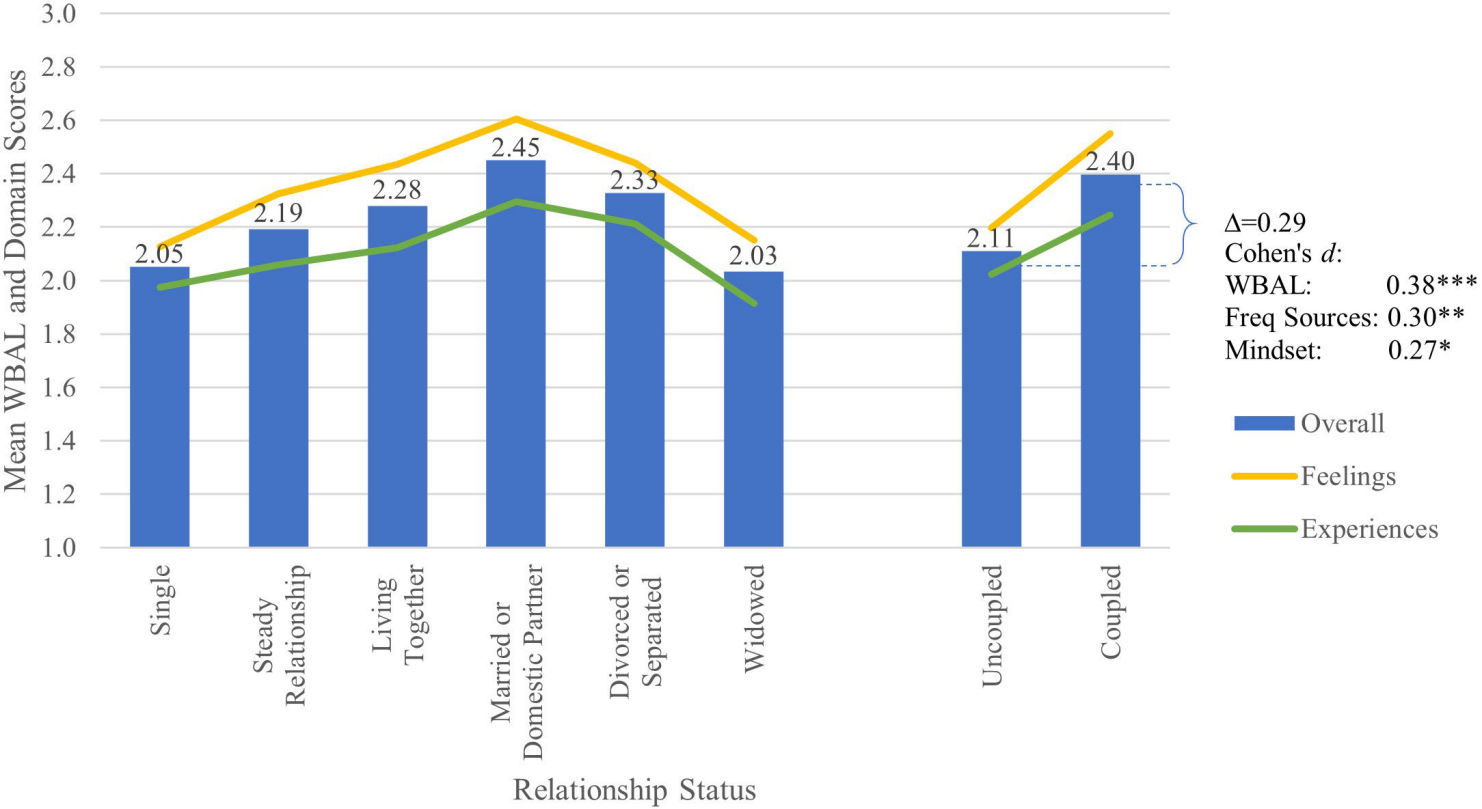
Mean WBAL and WBAL domain scores by relationship status, with Cohen’s *d* effect size for change in overall WBAL score, number of frequently positive experiences and feelings and mindset positivity. *p<0.05, **p<0.01, ***p<0.001. Symbols for Cohen’s *d* represent statistical significance of the specific Tukey’s HSD test.

As shown in **Figure 2** and **Table 4**, well-being increased as groups move from being uncoupled to more steady, committed relationships, with married respondents (or domestic partners) reporting the highest overall well-being and the most sources of well-being. As shown in **Table 5**, coupled respondents reported significantly higher well-being than uncoupled respondents (*d*=0.38) with more positive mindsets (*d*=0.27) and a wider range of sources of positive well-being (*d*=0.30). Coupled respondents reported significantly more frequent positive feelings overall (*d*=0.40) and across all WBAL factors (*d*’s from 0.22 to 0.44), particularly feelings of significance (*d*=0.44) and efficacy (*d*=0.37), with more frequent positive feelings across arousal levels (*d*=0.45). Coupled respondents also reported more frequent positive experiences (*d*=0.30), particularly more frequently spending time with a loving companion (*d*=1.13), providing for and nurturing others (*d*=0.23) and being calm and restful more often (*d*=0.54).

**Table 4:** Mean scores of WBAL overall, feelings, experiences and mindset positivity (Experiences less Feelings) by demographic category.

	Category	WBAL	Positive Experiences	Positive Feelings	Mindset Positivity
All Evaluable Subjects					
Gender Identification	Male	2.33	2.19	2.46	0.27
	Female	2.33	2.19	2.47	0.27
Age Range	Young Adults (20–29)	2.30	2.21	2.38	0.16
	Established Adults (30–44)	2.29	2.15	2.42	0.27
	Midlife Adults (45–60)	2.27	2.13	2.41	0.28
	Older Adults (61–69)	2.52	2.34	2.70	0.37
Annual Household Income	Lower Income ($25k-$50k)	2.33	2.22	2.43	0.21
	Middle Income ($50k-$75k)	2.32	2.21	2.42	0.21
	Higher Income ($75k-$100k)	2.35	2.18	2.52	0.34
	Highest Income ($100k+)	2.32	2.15	2.50	0.36
Employment Status	Unemployed	1.95	1.84	2.06	0.22
	Part-Time Employed	2.17	2.12	2.22	0.10
	Self-Employed	2.17	2.08	2.26	0.17
	Student	2.23	2.16	2.31	0.15
	Full-Time Employed	2.32	2.17	2.47	0.30
	Homemaker	2.50	2.38	2.63	0.25
	Retired	2.52	2.33	2.70	0.37
Relationship Status	Single	2.05	1.98	2.13	0.15
	Steady Relationship	2.19	2.06	2.33	0.27
	Living Together	2.28	2.12	2.44	0.31
	Married or Domestic Partnership	2.45	2.29	2.60	0.31
	Divorced or Separated	2.33	2.21	2.44	0.23
	Widowed	2.03	1.91	2.15	0.24
Parenting Status	No Children	2.16	2.08	2.23	0.15
	Single-Parent	2.23	2.09	2.37	0.28
	Co-Parent	2.30	2.14	2.46	0.32
	Two-Parent Household	2.42	2.26	2.58	0.32
	Parent with Children not Home	2.51	2.32	2.70	0.38

Color scale ranges from lowest values indicated as dark orange to highest values indicated as dark blue, with intermediate values in lighter shades proportional to position between lowest and highest values.

**Table 5:** With relationship status as independent variable, Cohen’s *d* effect sizes for domains, factors, and items.

Independent Variable: Relationship Status			Coupled vs. Uncoupled		Coupled				Uncoupled	
			*p*-value	Cohen’s *d*	*p*-value	Cohen’s *d*			*p*-value	Cohen’s *d*
Domain	Factor	Item	ANOVA	Coupled vs. Uncoupled	ANOVA	Married - Steady	Married – Living	Living - Steady	ANOVA	Divorced - Widow
Overall			0.0003 ***	0.38 ***	0.0462 *					
		Mindset Positivity	0.0113 *	0.27 *						
		Frequent Sources	0.0042 **	0.30 **	0.0407 *					
Experiences		0.0038 **	0.30 ***							
	Mind									
		Reflect Gratefully			0.0090 **		0.36 *			
	Connection		< 0.0001 ***	0.62 ***						
		Love Securely	< 0.0001 ***	1.13 ***						
	Purpose				0.0003 ***	0.71 ***		0.49 *		
		Contribute, Serve & Earn			0.0143 *	0.51 ***		0.53 *		
		Provide & Nurture	0.0228 *	0.23 *	< 0.0001 ***	0.80 ***		0.54 *		
		Kindness & Grace							0.0147 *	1.33 *
	Activity Range		0.0014 **	0.33 **						
		Calm & Restful	< 0.0001 ***	0.54 ***						
Feelings			0.0001 ***	0.40 ***						
	Openness		0.0082 **	0.27 **						
		Adventurous & Curious	< 0.0001 ***	0.45 ***						
		Harmonious & Attentive	0.0071 **	0.28 **	0.0480 *	0.39 *				
		Trusting & Safe	< 0.0001 ***	0.44 ***						
	Significance		< 0.0001 ***	0.44 ***						
		Belonging & Accepted	0.0319 *	0.22 *						
		Gentle & Loved	0.0123 *	0.26 *						
	Efficacy		0.0003 ***	0.37 ***	0.0038 **	0.47 *				
		Capable & Confident	0.0148 *	0.25 *						
		Considerate & Responsible	< 0.0001 ***	0.51 ***						
		Caring & Compassionate	< 0.0001 ***	0.43 ***						
	Wellness		0.0316 *	0.22 *						
		Vital & Strong	0.0010 ***	0.33 ***	0.0290 *					
		Satisfied & Fulfilled	< 0.0001 ***	0.43 ***	0.0016 **	0.48 **	0.34 *			
	Excitement Range		< 0.0001 ***	0.45 ***						
		Aware & Appreciative	0.0115 *	0.26 *						

Only comparisons with Cohen’s *d* > 0.2 and statistically significant Tukey’s HSD tests (*p* < 0.05) are shown (*p<0.05, **p<0.01, ***p<0.001).

Among coupled respondents, those who are married or living together reported significantly more frequent experiences of purposeful contribution than coupled respondents not yet living together (*d*’s from 0.49 to 0.71). Married respondents reported more frequently reflecting gratefully than other coupled respondents (*d*’s from 0.36 to 0.37), and feeling efficacious (*d*’s from 0.30 to 0.47), satisfied and fulfilled (*d*’s from 0.34 to 0.48) more frequently.

Among uncoupled respondents, there were no significant differences in overall well-being, positive feelings or positive experiences among single, divorced and widowed respondents. Divorced respondents reported the highest overall well-being among uncoupled respondents, comparable to those who are living with a companion and not yet married. No significant differences were observed between single and divorced or single and widowed subgroups.

#### 3.3.2 Parenting status

**Figure 3:**
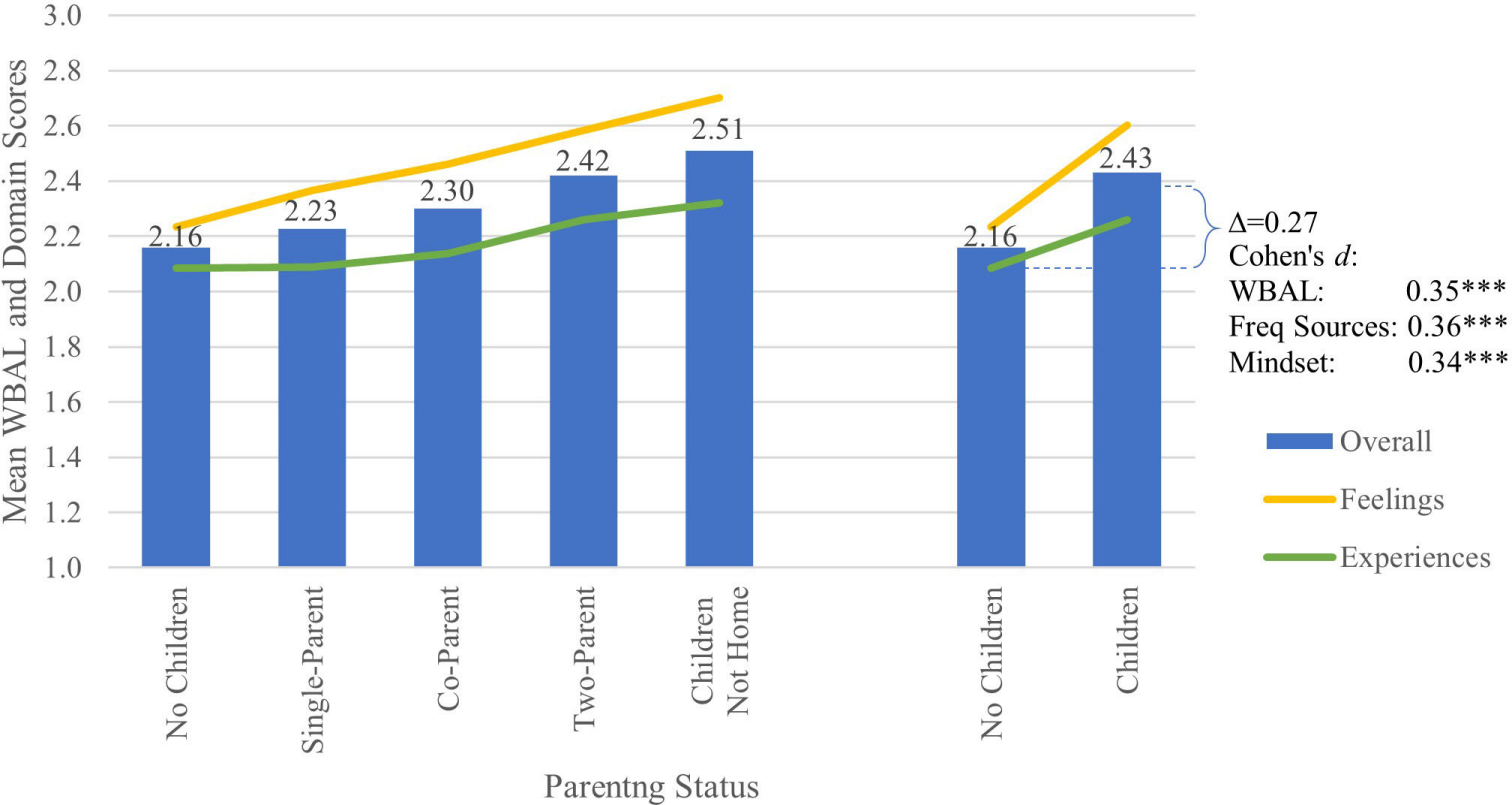
Mean WBAL and WBAL Domain scores by parenting status, with Cohen’s d effect size for change in overall WBAL score, number of frequently positive experiences and feelings, and mindset positivity. Note. ***p<0.001. Symbols for Cohen’s d represent statistical significance of the specific Tukey’s HSD test.

As shown in **Figure 3** and **Table 6**, being a parent was significantly associated with higher overall well-being (*d*=0.35), including positive feelings (*d*=0.41) and experiences (*d*=0.24). Parents reported significantly more positive mindsets (*d*=0.36) and more sources of positive well-being (*d*=0.34) than those without children. Parents reported more frequent positive feelings across a wider range of arousal levels than non-parents (*d*=0.44), with more frequent feelings of efficacy (*d*=0.45), significance (*d*=0.40) and wellness (*d*=0.36). Parents also reported a wider range of positive experience across activation levels (*d*=0.30) with more frequent social connection (*d*=0.25), including time with a loving companion (*d*=0.25), and more frequent purposeful experiences (*d*=0.34), including providing for and nurturing others (*d*=0.41).

**Table 6:** With parenting status as independent variable, Cohen’s *d* effect sizes for domains, factors, and items.

Independent Variable: Parenting Status			Parenting Status		Children		Active Parents	
			*p*-value	Cohen’s *d*	*p*-value	Cohen’s *d*	*p*-value	Cohen’s *d*
Domain	Factor	Item	ANOVA	Children – No Children	ANOVA	Not Home – Active Parent	ANOVA	Two – Single
Overall			0.0001 ***	0.35 ***				
		Mindset Positivity	0.0001 ***	0.36 ***				
		Frequent Sources	0.0003 ***	0.34 ***				
Experiences			0.0097 **	0.24 ***				
	Mind							
		Reflect Gratefully	0.0021 **	0.28 **				
	Connection		0.0071 **	0.25 **				
		Love Securely	0.0058 **	0.25 **			0.0057 **	0.63 **
	Purpose		0.0003 ***	0.34 ***				
		Provide & Nurture	< 0.0001 ***	0.41 **				
		Kindness & Grace	0.0477 *		0.0011 **	0.41 **		
	Activity Range		0.0014 **	0.30 **	0.0471 *	0.24 *		
		Mindful & Present	0.0001 ***	0.35 ***	0.0237 *	0.27 *		
		Calm & Restful	0.0092 **	0.24 **	0.0140 *	0.30 *		
Feelings			< 0.0001 ***	0.41 ***				
	Openness							
		Adventurous & Curious	0.0001 ***	0.38 ***				
		Harmonious & Attentive	0.0006 ***	0.32 ***	0.0134 *	0.30 *		
		Trusting & Safe	< 0.0001 ***	0.43 ***	0.0343 *	0.26 *		
	Significance		< 0.0001 ***	0.40 ***				
		Gentle & Loved	0.0090 **	0.24 **				
	Efficacy		< 0.0001 ***	0.45 ***				
		Capable & Confident	0.0008 ***	0.31 ***				
		Considerate & Responsible	0.0002 ***	0.35 ***			0.0146 *	0.63 *
		Caring & Compassionate	< 0.0001 ***	0.39 ***				
	Wellness		0.0001 ***	0.36 ***				
		Vital & Strong	< 0.0001 ***	0.39 ***				
		Satisfied & Fulfilled	< 0.0001 ***	0.43 ***				
		Peaceful & Serene	0.0003 ***	0.33 ***				
	Excitement Range		< 0.0001 ***	0.44 ***	0.0243 *	0.27 *		
		Joyful & Confident	0.0007 ***	0.32 ***				
		Aware & Appreciative	0.0004 ***	0.33 ***	0.0174 *	0.29 *		
		Content & Peaceful	0.0013 **	0.30 **				

Only comparisons with Cohen’s *d* > 0.2 and statistically significant Tukey’s HSD tests (*p* < 0.05) are shown (*p<0.05, **p<0.01, ***p<0.001).

Parents whose children are no longer at home reported a significantly wider range of positive experiences across activation levels than active parents (*d*=0.24). They reported more frequent experiences of treating others with kindness and grace (*d*=0.41). Additionally, they experienced more frequent positive feelings across arousal levels (*d*=0.27), especially feelings of harmony and attentiveness (*d*=0.30), trust and safety (*d*=0.26) and awareness and appreciation (*d*=0.29).

The ability to share parenting responsibilities positively affected two key sources of well-being. In addition to reporting more frequently spending time with a loving trusted companion (*d*=0.63), respondents in two-parent households felt considerate and responsible (*d*=0.63) more frequently than single parents. No significant differences were observed between single parents and co-parents.

#### 3.3.3 Employment status

**Figure 4:**
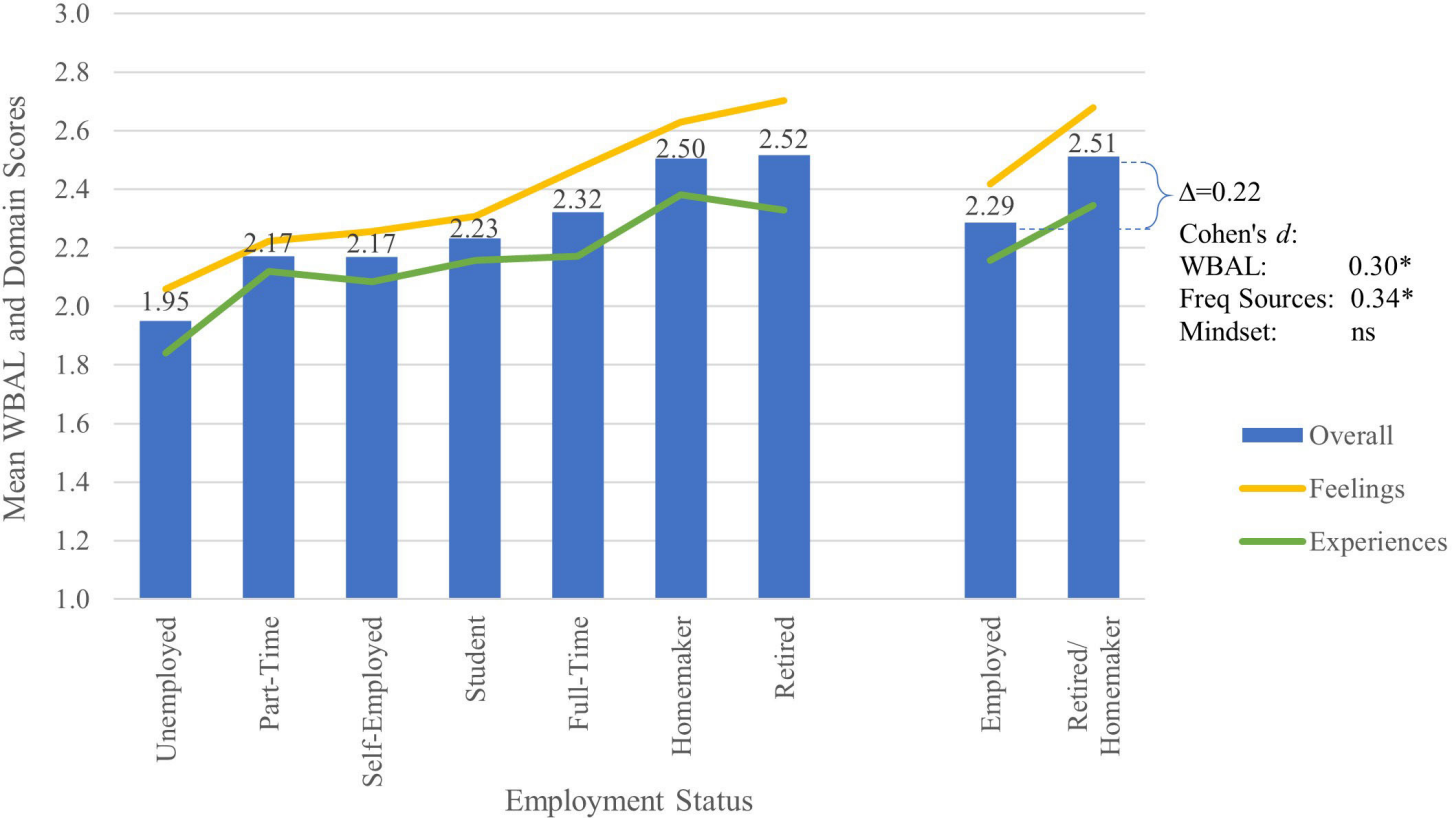
Mean WBAL and WBAL Domain scores by employment status, with Cohen’s d effect size for change in overall WBAL score and number of frequently positive experiences and feelings. Note. * p<0.05; ns, non-significant. Symbols for Cohen’s d represent statistical significance of the specific Tukey’s HSD test.

Employment status was significantly associated with well-being in this respondent sample. Retirees and homemakers reported the highest overall well-being, as shown in **Figure 4**, and significantly higher well-being than employed respondents (*d*=0.30), as shown in **Table 7**. Among those available to work, well-being trended upward with the degree of employment. Unemployed respondents seeking a job reported the lowest well-being, while full-time employees reported the highest. Part-time, self-employed and students reported intermediate levels of well-being.

**Table 7:** With employment status as independent variable, Cohen’s *d* effect sizes for domains, factors, and items.

Independent Variable: Employment Status			Employment					Full/Part/Self-Employed		
			*p*-value	Cohen’s *d*				*p*-value	Cohen’s *d*	
Domain	Factor	Item	ANOVA	Ret/Hom – Emp	Ret/Hom – Stud	Ret/Hom - Unem	Emp – Unem	ANOVA	Full - Part	Full - Self
Overall			0.0145 *	0.30 *						
		Frequent Sources	0.0074 **	0.34 *						
Experiences			0.0269 *	0.27 *						
	Mind		0.0484 *							
		Reflect Gratefully	0.0414 *	0.30 *						
	Connection		0.0246 *							
		Love Securely	0.0137 *		0.72 *					
	Purpose									
		Provide & Nurture	0.0031 **	0.35 **	0.64 *					
	Activity Range									
		Mindful & Present	0.0020 **	0.42 ***						
		Calm & Restful	0.0071 **	0.29 *	0.67 *			0.0194 *		0.39 *
Feelings			0.0148 *	0.29 *						
	Openness									
		Adventurous & Curious	0.0003 ***	0.42 ***		0.80 *				
		Harmonious & Attentive	< 0.0001 ***	0.56 ***						
		Trusting & Safe	0.0004 ***	0.41 **		0.80 *		0.0143 *		
	Efficacy							0.0148 *	0.43 *	
		Considerate & Responsible						0.0430 *		
	Wellness									
		Vital & Strong	0.0441 *					0.0236 *		
		Satisfied & Fulfilled						0.0014 **	0.55 **	
	Excitement Range		< 0.0001 ***	0.53 ***	0.68 *			0.0473 *		
		Joyful & Confident	0.0176 *			1.10 **	1.02 *			
		Content & Peaceful	0.0386 *	0.29 *						

Only comparisons with Cohen’s *d* > 0.2 and statistically significant Tukey’s HSD tests (*p* < 0.05) are shown (*p<0.05, **p<0.01, ***p<0.001).

Compared to employed respondents, retirees and homemakers reported significantly more sources of well-being (*d*=0.34), more frequent positive experiences (*d*=0.27) and more frequent positive feelings (*d*=0.29) across a wider range of arousal levels (*d*=0.53). Unemployed respondents reported significantly less frequent feelings of joy and confidence than those employed (*d*=1.02) or retirees or homemakers (*d*=1.10). No significant differences were observed between retired and home-maker respondents.

Among employed respondents, full-time employees reported significantly more frequent feelings of efficacy (*d*=0.43) and fulfillment (*d*=0.55) than part-time employees. Full-time employees also reported more frequent feelings of calm and restfulness (*d*=0.39) than self-employed respondents. No significant differences were observed between part-time and self-employed respondents. Students reported overall well-being similar to employed respondents, with no significant differences observed between these groups.

#### 3.3.4 Age

**Figure 5:**
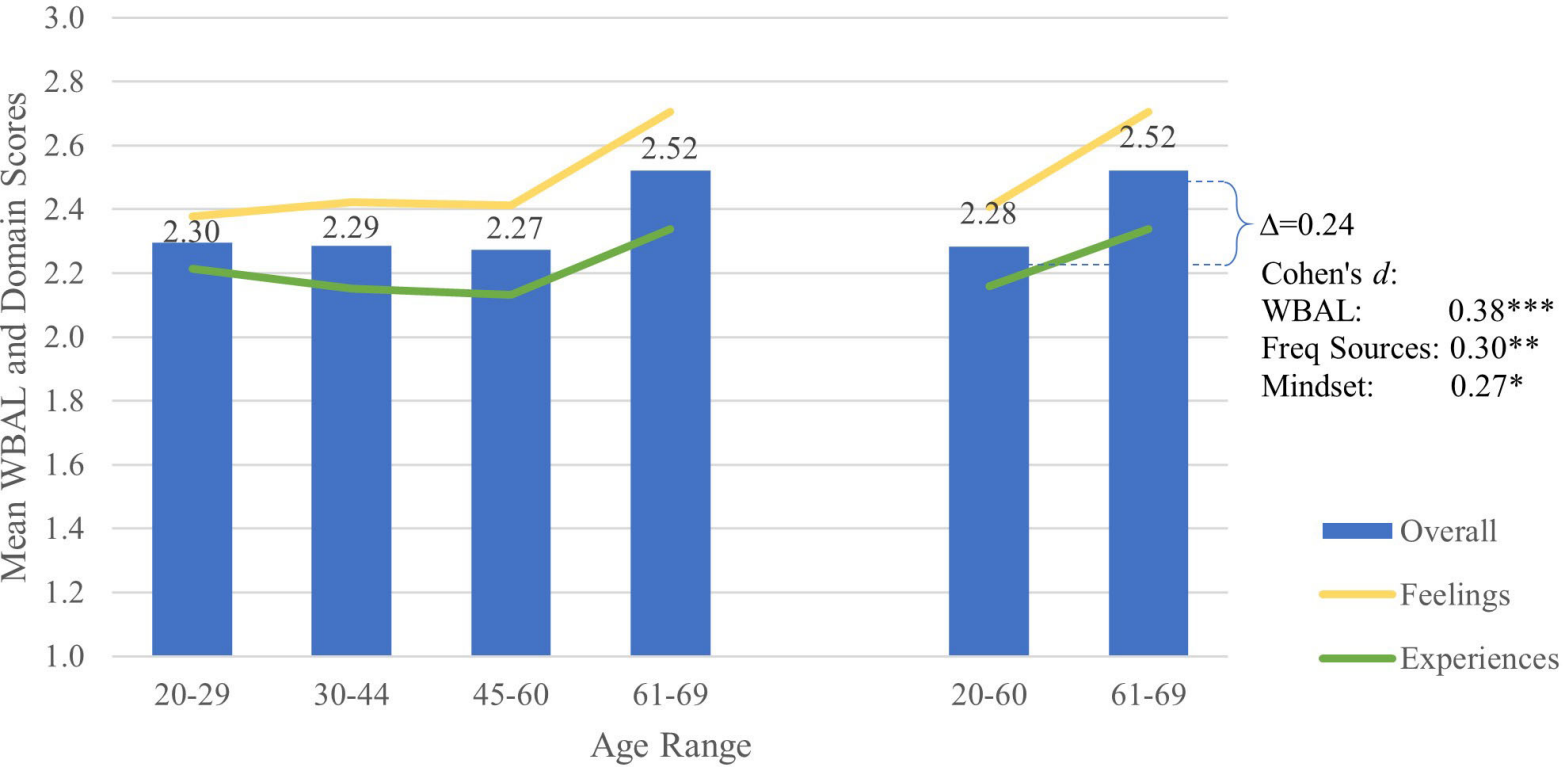
Mean WBAL and WBAL domain scores by age, with Cohen’s *d* effect size for change in overall WBAL score, number of frequently positive experiences and feelings and mindset positivity. *p<0.05, **p<0.01, ***p<0.001. Symbols for Cohen’s *d* represent statistical significance of the specific Tukey’s HSD test.

As shown in **Table 8**, age was a significant predictor of differences in overall WBAL score, as well as frequency of positive experiences and feelings of well-being. Respondents aged 20 to 60 years old reported similar positive well-being, with no significant differences in any domain or factor. However, reported well-being increased significantly in the older adult subgroup (aged 61-69), primarily due to a rise in the frequency of positive feelings (*d*=0.33), as illustrated in **Figure 5** and **Table 4**.

**Table 8:** With age as independent variable, Cohen’s *d* effect sizes for domains, factors, and items.

Independent Variable: Age			Older Adults/All Other		All Other Age Groups	
			*p*-value	Cohen’s *d*	*p*-value	Cohen’s *d*
Domain	Factor	Item	ANOVA	Older Adults – All Others	ANOVA	Young - Midlife Adults
Overall			0.0068 **	0.31 **		
		Frequent Sources	0.0030 **	0.34 **		
Experiences			0.0218 *	0.27 *		
	Body					
		Move Regularly			0.0080 **	0.40 **
	Purpose		0.0364 *	0.25 *		
		Kindness & Grace	0.0160 *	0.29 *		
	Activity Range		0.0192 *	0.27 *		
		Active & Engaged			0.0185 *	0.36 *
		Mindful & Present	0.0017 **	0.37 **		
Feelings			0.0034 **	0.33 **		
	Openness		0.0006 ***	0.25 ***		
		Harmonious & Attentive	0.0494 *	0.23 *		
	Significance					
		Proud & Mattering	0.0099 **	0.30 **	0.0205 *	0.37 *
		Belonging & Accepted	0.0236 *	0.26*		
	Efficacy		0.0258 *	0.25 *		
		Capable & Confident	0.0295 *	0.25 *		
		Considerate & Responsible	0.0071 **	0.31 **		
		Caring & Compassionate	0.0416 *	0.24 *		
	Wellness		0.0073 **	0.31 **		
		Vital & Strong	0.0018 **	0.36 **		
		Satisfied & Fulfilled	0.0056 **	0.32 **		
		Peaceful & Serene	0.0079 **	0.31 **		
	Excitement Range		0.0012 **	0.38 **		
		Joyful & Confident	0.0067 **	0.31 **		
		Aware & Appreciative	0.0010 ***	0.46 ***		
		Content & Peaceful	0.0217 *	0.27 *		

Only comparisons with Cohen’s *d* > 0.2 and statistically significant Tukey’s HSD tests (*p* < 0.05) are shown (*p<0.05, **p<0.01, ***p<0.001).

Older adults experienced a greater increase in positive feelings compared to positive experiences. While older adults reported higher mindset positivity than other age groups, this trend did not reach significance (*d*=0.22, *p*=0.056). Nevertheless, older adults reported significantly more frequent positive feelings across a wider range of arousal levels (*d*’s from 0.27 to 0.46). They also reported more frequent positive experiences (*d*=0.27) across a wider range of activation levels (*d*=0.27) relative to younger age groups.

Midlife adults reported the lowest overall WBAL scores. While they more frequently provided for and nurtured others than young adults (*d*=0.30), they were less frequently physically active (*d*=0.40) and engaged (*d*=0.36), and less frequently felt proud and that their lives mattered (*d*=0.37) compared to young adults. No significant differences were observed between midlife and established adults, or between established and young adults.

#### 3.3.5 Gender

As shown in **Table 4**, there were no differences reported between genders in overall WBAL, mindset positivity or overall frequency of positive experiences or positive feelings. Men reported being physically active more frequently than women (ANOVA *p*=0.018; *d*=0.22, *p*<0.05), whereas women reported connecting with friends and family more often than men (ANOVA *p*=0.003; *d*=0.27, *p*<0.01).

#### 3.3.6 Annual household Income

**Figure 6:**
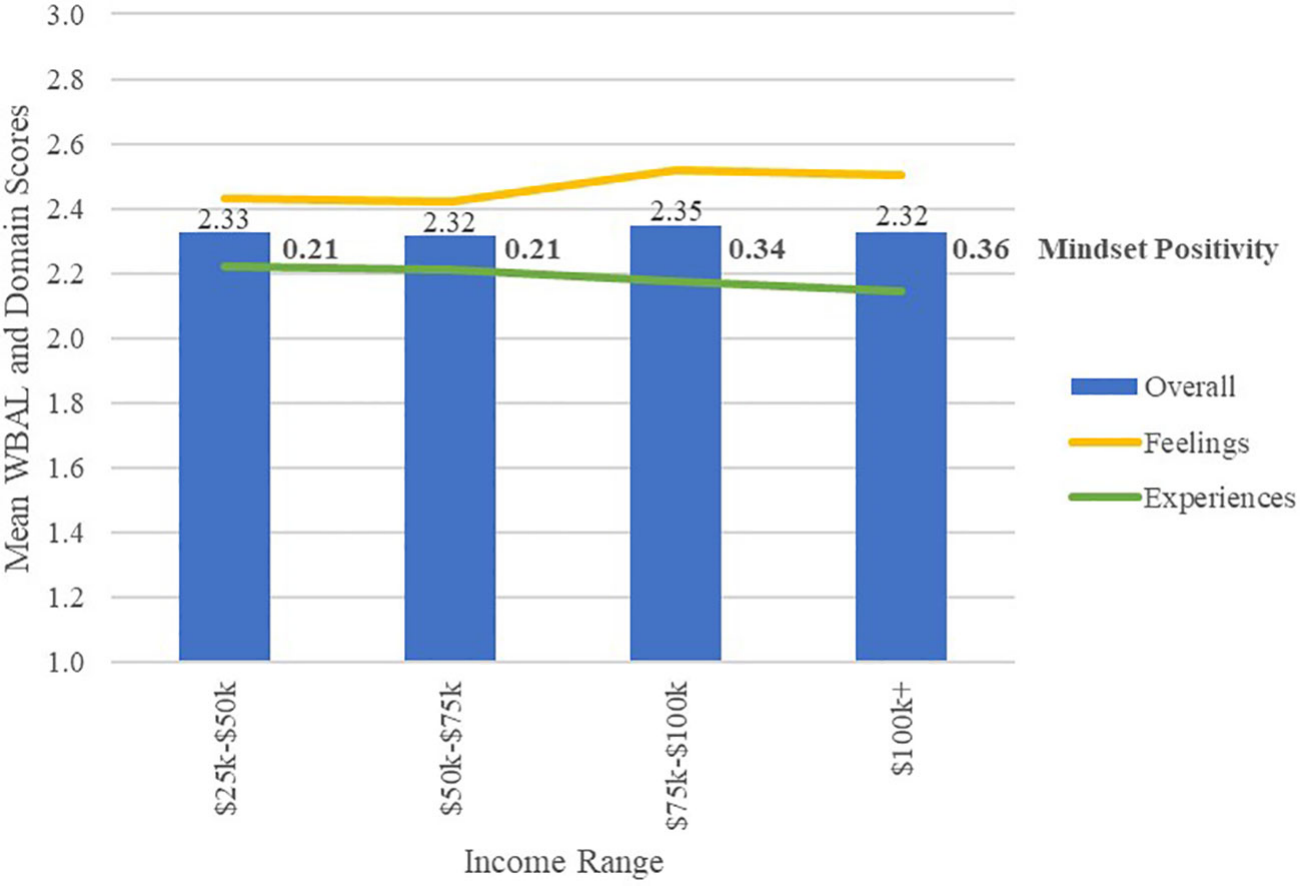
Mean WBAL, WBAL domain scores and mindset positivity by annual household income.

As measured by the WBAL instrument, respondents reported only small differences in well-being by income bracket, as illustrated in **Figure 6** and **Table 4**. Household income showed a significant effect on mindset positivity, with a non-significant trend of increasing positivity from lower to higher income groups, and a moderate effect size between the highest and lowest income groups (*d*=0.29, ns). However, this trend towards improved mindset positivity did not translate to higher overall well-being as measured by WBAL, because respondents in higher income brackets reported a lower frequency of positive experiences.

## 4 Discussion

This study applied the WBAL Assessment to understand the impact of different indicators of well-being on overall well-being and sources of well-being across population subgroups. Life situations such as being coupled, a parent or retired, showed a more significant impact on overall well-being than age, gender or household income. The largest underlying sources of these differences in well-being were social connection and purposeful contributions, with associated feelings of significance and efficacy.

The findings also point to the importance of time affluence and agency for positive well-being, and suggest an integrated model of intrinsic motivation and the importance of motivation, meaning and mattering for human flourishing. Finally, this study suggest that having a wide range of sources of positive well-being across activation and arousal levels works together with a positive mindset to increase well-being.

### 4.1 Connection and purposeful contributions

Life situations that increase the frequency of social connection and purposeful contributions to others’ well-being, coupled with frequent feelings of efficacy and significance, had the most significant positive impact on well-being. Respondents in life situations that create more frequent opportunities for positive experiences of human connection reported higher well-being and more sources of well-being than those with fewer opportunities for social connection. And whether considering relationship or parenting status, feelings of significance and efficacy closely corresponded with experiences of social connection and purposeful contribution, suggesting that these factors work together to enhance well-being.

Being coupled, and particularly being married, improved well-being broadly for respondents in this study, consistent with prior research (14–19). This improvement corresponded with the most positive mindset, most frequently feeling efficacious, significant and well, as well as the most frequent experiences of trusted loving companionship and providing for and nurturing others. Progression of relationship commitment from steady relationship to living together to marriage was associated with significant increases in the frequency of experiences entailing purposeful contribution to others’ well-being.

Parenting had a strong influence on well-being, and increased with greater sharing of parenting responsibilities, also consistent with prior research (20–22). Parents reported significantly more frequent experiences of social connection and purposeful contribution with more frequent feelings of significance and efficacy.

### 4.2 Time affluence and agency

Differences in well-being between respondents in different parenting and employment situations reinforce the importance of having a combination of time affluence and agency for well-being.

Time affluence appears to play an important role in fostering positive well-being across a range of experience activation and feelings arousal levels. Parents with children not at home reported the widest range of activation and arousal levels. And homemakers and retirees not working by choice reported the highest overall well-being among employment status categories, with the widest range of positive sources of well-being across activation and arousal levels. This is consistent with previous research showing that later in life (ages 60 and up), working under pressure reduces subjective well-being, and those who do not work enjoy a higher level of life satisfaction (64).

In contrast, unemployment, part-time employment and self-employment reduced the frequency of multiple sources of well-being, consistent with prior research (23–25). Although a small proportion of the total study sample, unemployed respondents reported the lowest well-being across all subgroups studied, with significantly fewer sources of positive well-being than employed respondents. The lower well-being observed among under- and unemployed respondents actively seeking work, relative to retirees and homemakers choosing not to work, points to the importance for well-being of having agency over whether and how much we work.

### 4.3 Motivation, meaning and mattering

Social connection, purpose, feelings of efficacy, and agency are key drivers of intrinsic motivation and well-being. Self-determination theory establishes competence, autonomy, and relatedness as essential needs for intrinsic motivation and well-being (65). And the autonomy, mastery, purpose framework posits that these are three fundamental factors for intrinsic motivation (66).

Furthermore, feeling purpose and significance are important components of meaning (67, 68), and closely corresponded with overall positive well-being in this study. Having a sense of purpose with coherent life goals provides personal meaning that can be a renewable source of motivation and engagement (69).

Feelings that our lives matter depend upon social relationships that entail reciprocally adding value and feeling valued (70), suggesting a bidirectional effect between social connection and making purposeful contributions. Meaningfulness, which involves being a “giver” to others over time (39), and a sense of belonging and connectedness are associated with more positive well-being (71). The findings from this study therefore suggest an integrated model of the roles of motivation, meaning and mattering for positive well-being that encompasses five closely inter-related factors: autonomy; social connection; purposeful contributions; feelings of efficacy, competence and mastery; and feelings of significance and mattering.

### 4.4 Well-being breadth and mindset positivity

This study suggests that the breadth of sources of well-being, with a balance across activity and arousal levels, works together with a positive mindset to increase overall well-being. Although breadth of well-being sources appears to be a primary driver of positive well-being, a positive mindset can offset life situations or demographic indicators associated with less frequent positive experiences.

Life situations associated with higher overall well-being, such as being coupled, a parent or retired, broadly improved well-being. This improvement was primarily due to more frequent positive experiences with positive feelings spanning a wider range of arousal levels. For every comparison with a significant difference in overall WBAL, significant differences were also observed in the number of frequent sources of positive well-being, as well as the frequency and range of positive feelings from low to high arousal levels, confirming the importance of a variety of experiences and feelings for positive well-being.

An age-related positivity effect was observed between older adults (60-69 years old) and all other age groups, consistent with prior research indicating that this effect begins in the mid-50’s to early 60’s (72). This increase in well-being among older adults was primarily driven by significantly more frequent and a broader range of positive feelings of well-being, with a moderate trend towards increased mindset positivity, as older adults also reported more frequent positive experiences.

Being coupled or a parent was associated with increased mindset positivity and overall well-being. Although mindset positivity was significantly associated with higher income, annual household income had no independent effect on overall well-being in this study because reported frequency of positive experiences trended downward with increased income, suggesting that higher earners felt more positively about their lives, whereas those with lower income engaged more frequently in positive experiences.

## 5 Strengths and limitations

Unlike previous studies which often only assess feelings of well-being or focus on isolated aspects of well-being, the WBAL Assessment integrates both experienced and felt contributors to well-being. The WBAL Model provides a unique and comprehensive evaluation of positive well-being across various demographic groups and life situations by simultaneously assessing a wide range of positive experiences and feelings across a full range of activation and arousal levels. This enables the investigation of relationships among the frequency and breadth of positive experiences and feelings, as well as mindset positivity and range of feelings arousal levels, providing a better understanding of the effects of demographics, lifestyle situations and other subgroup characteristics on well-being.

The WBAL Assessment’s ability to measure the number of frequent sources of positive well-being, and feelings with positive affect across a range of arousal levels, provides additional insights into the importance of having a breadth of positive experience and feelings with a balance of arousal levels for overall well-being. These measures not only serve as useful summaries of overall well-being but may also offer promising targets for interventions aimed at enhancing well-being. For example, increasing the breadth of frequent positive experiences and feelings while expanding the range of positive feelings across low, moderate and high arousal levels could be effective generalizable strategies for improving well-being.

### 5.1 Guiding interventions to enhance well-being

A deeper understanding of the interactions between positive experiences and feelings can guide the design of interventions to improve well-being. This study demonstrated that social connection and purposeful contributions to others’ well-being corresponded with feelings of significance and efficacy, each of which are important drivers of intrinsic motivation and meaning. This suggests that interventions focused on increasing social connection and tangible contributions to others may be more effective if they also enhance feelings of significance and efficacy.

The findings from this study have important practical implications for community initiatives, workplace practices, and educational programs. Programs that encourage social connections, provide opportunities for purposeful contributions, and support positive mindsets can improve well-being. In workplace settings, promoting social interactions, providing employee agency and recognizing contributions can enhance overall well-being. Educational programs that integrate well-being practices into their curricula, particularly those that support students’ search for a sense of purpose, efficacy and meaning, can help students develop life-long skills for building a fulfilling life. These applications demonstrate the practical value of the WBAL model for designing interventions that target specific sources of well-being.

Findings can also guide the design and implementation of intervention programs for targeted subgroups. For example, individuals without children or a companion as well as those in lower-income households, may benefit from mindset-based interventions, whereas those in higher income households may benefit from introducing more positive experiences into their lives.

This study identified physical self-care and social connection as the least frequently experienced sources of well-being, with differences between genders. While women and men in this study reported the same overall well-being, women reported being physically active less frequently, suggesting movement-based interventions, while men reported less frequent social interactions, suggesting interventions to increase social connection.

The WBAL Assessment is a useful instrument for measuring positive well-being and assessing changes in the nature, frequency and range of feelings of positive well-being. This enables deeper understanding of these inter-relationships and allows for more tailored interventions for individuals and targeted populations.

### 5.2 Enabling broadly reliable replication

This study replicates and provides a deeper understanding of several previously reported effects, including: the fundamental importance of positive human connection and feelings of significance for well-being; increased well-being associated with more committed companionship; loss of well-being across multiple dimensions accompanying under- and unemployment; age-related positivity effects; similarity of self-reported well-being between genders with gender differences in physical activity and social connection; the role of mindset positivity for enhancing well-being; and the importance of broadening sources of well-being for overall well-being.

By replicating a wide range of prior well-being research studies across different subgroups using a single well-being assessment that can be completed quickly, this study further validates the WBAL Assessment as a useful instrument to evaluate positive well-being across subgroups. Furthermore, it effectively identifies discrete underlying sources of well-being. Through the replication of results from previous research, this study reinforces the validity of established relationships, such as the impact of relationship status, parenting, and employment on well-being, while enabling comparison of the significance of these effects relative to other indicators such as age, gender and income. Moreover, the WBAL assessment provides novel insights into the complex interactions among different sources of well-being across different life situations and their combined influence on overall well-being. The replication and extension of these findings support the robustness of the WBAL model and its potential to enhance the understanding of well-being across diverse populations.

### 5.3 Limitations and further research

While this study provides valuable insights into the sources of well-being, several limitations should be noted. The sample is limited to English speakers in the United States with incomes over $25,000 and ages between 20 and 69 years, which may not be representative of the broader population. Additionally, the study relies on self-reported data, which can be subject to biases such as social desirability and recall bias.

Future research should consider a more diverse sample, including individuals from different cultural and socioeconomic backgrounds, as well as younger and older age groups and those with different health statuses. Other interesting independent variables to assess include non-binary gender identity, sexual preferences and non-traditional relationship models, as well as household net worth and homeownership. Longitudinal studies would also be beneficial to understand the causality of the relationships observed and how well-being evolves over time.

Studies with larger sample sizes or balanced sampling methods to confirm non-significant trends observed in this study may be informative. For example, this study observed non-significant trends that may suggest a broad negative impact of unemployment across well-being sources, increased well-being with more sharing of parenting responsibilities, and increased frequency of positive experiences and positivity practices among lower income respondents.

The study observed age subgroups at a single point in time without investigating changes in individuals over time. Therefore, age-related findings should not be considered definitive of past or future life cycles of individuals progressing through age groups. Some of the differences seen across age groups may indeed be due to natural progression of well-being over the course of a life, whether driven by biological, psychological or long-lasting social factors. However, differences in well-being observed between age groups may be generational, and thus affected by influences such as historical events, societal trends, macroeconomics or changes in technology.

Importantly, this study does not address causality of relationships among subgroups, well-being or categorical sources of well-being. Many of the observed associations are likely to have reciprocal causality and many of the indicators correlate strongly, requiring further research to understand underlying causes, mediating factors and potential benefits of interventions for well-being. Larger sample sizes will also be required to better understand interaction effects across well-being indicators.

## 6 Conclusions

Life situations, including relationship, parenting and employment status, had a more broad and significant effect on wellbeing than age, gender or income. Across life situations, purposeful contribution and social connection, with associated feelings of efficacy and significance were key drivers of differences in well-being. Mindset positivity and variety of positive experiences and feelings correspond closely with overall well-being. Findings from this study can help guide the design and implementation of intervention programs to improve well-being for individuals and targeted subgroups, demonstrating the utility of the WBAL Assessment to evaluate discrete modifiable sources of positive well-being.

## Figures and Tables

**APPENDIX FIGURE 1:**
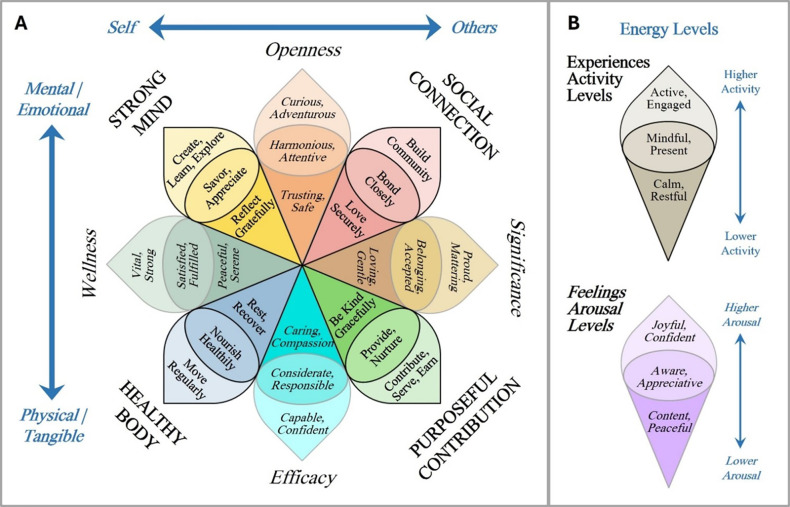
**(A)** The WBAL model. factors within the experiences domain are denoted with all capitalized letters and factors within the Feelings domain are denoted with only the first letter capitalized. **(B)** Activation and *arousal energy levels within WBAL experiences and feelings* domains, respectively. Reprinted from 'Well-being balance and lived experiences assessment: a valid, comprehensive measure of positive well-being' by McDermott et al (2024) (58), CC-BY 4.0, https://www.frontiersin.org/journals/psychology/articles/10.3389/fpsyg.2024.1396543/full.

## Data Availability

The original contributions presented in the study are included in the article/**Supplementary Material**. Further inquiries can be directed to the corresponding author.

## Appendix A: well-being balance and lived experiences (WBAL) model

Drawing together findings across positive psychology and well-being fields of research, the WBAL Model posits that our subjective sense of well-being arises from positive life experiences including caring for ourselves mentally and physically by attending to our minds and bodies, while engaging with others emotionally and tangibly by nurturing positive social relationships and engaging in purposeful activities that contribute to others’ well-being. Additionally, the WBAL Model aims to encompass the full range of hedonic and eudaimonic positive experiences and feelings, which balances mental and physical activity and stimulation with savoring and mindful engagement, and rest and reflection. Each item of positive experiences corresponds with a body of evidence supporting the positive impact of these activities and experiences on well-being.

A study validating the WBAL Model (58), which measures individuals’ recent frequency of a range of positive experiences and positive feelings, demonstrated that more frequent positive experiences overall correspond with more frequent positive feelings overall, and that together, these correspond closely with an individual’s self-reported overall well-being. Furthermore, having more categories of frequent positive experiences and feelings corresponds with increased overall well-being, consistent with an upward spiral of positivity as predicted by the broaden and build theory of positive emotions (47).

The WBAL Model is illustrated in **Appendix Figure 1**, Panel A as a lotus flower (WBAL Lotus) that interweaves items of positive experiences with items of related positive feelings. The model has two (2) domains of Experiences and Feelings, with eight (8) factors including four (4) Experiences factors (Body, Mind, Connection, and Purpose) and four (4) Feelings factors (Wellness, Openness, Significance, and Efficacy), represented as separate petals of the lotus flower. Positive experience items have been demonstrated to correspond more closely with adjacent than distant positive feelings items, as represented in the WBAL Lotus.

Within each factor, energy levels denote relative Activation levels of Experiences and Arousal levels of Feelings, with lower activation and arousal levels at the center and higher activation and arousal levels along the outside of the petals of the WBAL Lotus. As shown in **Appendix Figure 1**, Panel B, for Experiences these energy levels are referred to as Activation levels: Active/Engaged, Mindful/Present, and Calm/Restful. For Feelings these Energy levels are referred to as Arousal levels: Joyful/Confident, Aware/Appreciative, and Content/Peaceful. Each discrete energy level within a factor is a distinct source of well-being that corresponds to an item on the WBAL Assessment. Additionally, the WBAL Assessment includes three items of each energy level to assess the overall range of Experience Activation levels and three corresponding items to assess the overall range of Feelings Arousal levels.
